# Tyrosol May Prevent Obesity by Inhibiting Adipogenesis in 3T3-L1 Preadipocytes

**DOI:** 10.1155/2020/4794780

**Published:** 2020-12-09

**Authors:** Francesca Pacifici, Carolina Lane Alves Farias, Silvia Rea, Barbara Capuani, Alessandra Feraco, Andrea Coppola, Caterina Mammi, Donatella Pastore, Pasquale Abete, Valentina Rovella, Chiara Salimei, Mauro Lombardo, Massimiliano Caprio, Alfonso Bellia, Paolo Sbraccia, Nicola Di Daniele, Davide Lauro, David Della-Morte

**Affiliations:** ^1^Department of Systems Medicine, University of Rome Tor Vergata, Rome 00133, Italy; ^2^PhD School of Applied Medical-Surgical Sciences, University of Rome Tor Vergata, 00133 Rome, Italy; ^3^CAPES Scholarship (Proc No. BEX 013069/2013-06), CAPES Foundation, Ministry of Education of Brazil, Brasília, DF 70040-020, Brazil; ^4^Laboratory of Cardiovascular Endocrinology, IRCCS San Raffaele Pisana, 00166 Rome, Italy; ^5^Department of Translational Medical Sciences, University of Naples “Federico II”, 80138 Naples, Italy; ^6^Department of Medical Sciences, Fondazione Policlinico Tor Vergata, 00133 Rome, Italy; ^7^PhD School of Neuroscience, University of Rome Tor Vergata, 00133 Rome, Italy; ^8^Department of Human Sciences and Quality of Life Promotion, San Raffaele Roma Open University, 00166 Rome, Italy; ^9^Department of Neurology and Evelyn F. McKnight Brain Institute, Miller School of Medicine, University of Miami, Miami, FL, USA

## Abstract

Tyrosol (TR), a major polyphenol found in extra virgin olive oil (EVOO), exerts several antioxidant effects. However, only scarce evidences are present regarding its activity on adipocytes and obesity. This study evaluated the role of TR in adipogenesis. Murine 3T3-L1 preadipocytes were incubated with TR (300 and 500 *μ*M), and TR administration inhibited adipogenesis by downregulation of several adipogenic factors (leptin and aP2) and transcription factors (C/EBP*α*, PPAR*γ*, SREBP1c, and Glut4) and by modulation of the histone deacetylase sirtuin 1. After complete differentiation, adipocytes treated with 300 and 500 *μ*M TR showed a reduction of 20% and 30% in lipid droplets, respectively. Intracellular triglycerides were significantly reduced after TR treatment (*p* < 0.05). Mature adipocytes treated with TR at 300 and 500 *μ*M showed a marked decrease in the inflammatory state and oxidative stress as shown by the modulation of specific biomarkers (TNF, IL6, ROS, and SOD2). TR treatment also acted on the early stage of differentiation by reducing cell proliferation (~40%) and inducing cell cycle arrest during Mitotic Expansion Clonal (first 48 h of differentiation), as shown by the increase in both S1 phase and p21 protein expression. We also showed that TR induced lipolysis by activating the AMPK-ATGL-HSL pathway. In conclusion, we provided evidence that TR reduces 3T3-L1 differentiation through downregulation of adipogenic proteins, inflammation, and oxidative stress. Moreover, TR may trigger adipose tissue browning throughout the induction of the AMPK-ATGL-UCP1 pathway and, subsequently, may have promise as a potential therapeutic agent for the treatment and prevention of obesity.

## 1. Introduction

Obesity is a pandemic public health problem, especially in developing countries, where the prevalence is increasing exponentially in the last decades [1]. Obesity is considered the leading cause of risk for cardiovascular disease (CVD) and metabolic diseases [2]. It is a complex disorder with multifactorial etiology, characterized by an imbalance between energy intake and energy expenditure, which, in turn, induces a pathological growth of adipose tissue [3, 4]. Adipose tissue is not a passive reservoir for energy storage. It takes, in fact, an important role in the regulation of energetic and endocrine homeostasis, by controlling the release of anti- and proinflammatory adipokines, like leptin [5]. Alterations in adipocyte biology, typical of obesity state, lead to systemic inflammation, increase in chronic oxidative stress, and obesity-related diseases [3]. Thus, strategies able to reduce the pathological inflammatory state and oxidative stress mediated by adipocyte dysfunction may be helpful in counteracting the epidemic burden of obesity.

Among the main strategies proposed to prevent obesity in adults, including increase in physical activity [6] and caloric restriction [7], the increase in consumption of food enriched with anti-inflammatory and antioxidant compounds, such as polyphenols [8], recently raised attention. Polyphenols, such as resveratrol and quercetin, which are secondary metabolites of plants, have been reported to exert antiobesity effects by acting at different levels on adipocyte maturation [9]. In particular, they regulate preadipocyte proliferation, block adipogenesis, and induce apoptosis [10]. Polyphenols are present in the Mediterranean diet, which is a plant-based diet that became a cornerstone for the prevention of chronic diseases [11]. The Mediterranean diet, compared to other dietary regimens, exerts more beneficial health effects just for the presence of a high intake of unique polyphenols contained in the extra virgin olive oil (EVOO), among others [11, 12]. EVOO contains more than 30 polyphenolic compounds; among those, in particular, hydroxytyrosol (HT) and Tyrosol (TR) are the most absorbed and, thus, bioavailable in humans [13, 14]. TR is absorbed after ingestion in a dose-dependent manner via passive diffusion, and in humans, the absorption is as high as 55–66% since it becomes conjugated to glucuronic acid and excreted in urine as glucuronides. Therefore, a high concentration of TR is suggested to exert its effect [15]. A recent study reported that HT and oleuropein (another EVOO polyphenolic compound) prevent adipogenesis and reduce preadipocyte proliferation [16], suggesting that these phytochemicals might prevent obesity. Another interesting study already demonstrated that high doses of TR (0.1–1 mg/mL) are able to reduce differentiation of preadipocytes [17]. Nevertheless, to the best of our knowledge, at the moment, no data regarding possible mechanisms involved in the beneficial role of TR in adipogenesis and therefore obesity are present. In the present study, we sought to evaluate the direct effects of TR on adipogenesis by using a murine 3T3-L1 preadipocyte cell line.

## 2. Materials and Methods

### 2.1. Cell Culture and Adipocyte Differentiation

Mouse 3T3-L1 cells were gently provided by Dr. Massimiliano Caprio (San Raffaele Open University, Rome, Italy) and cultured in Dulbecco's modified Eagle's medium (DMEM) (Thermo Fisher Scientific, Waltham, Massachusetts, USA), containing 10% fetal calf serum (ATCC, Manassas, Virginia, USA) and 1% penicillin/streptomycin (Thermo Fisher Scientific) and maintained at 37°C in a humidified, 5% CO_2_ atmosphere. Cells were differentiated 2 days after reaching confluence, then were stimulated with DMEM (4.5 g/L glucose) containing 10% fetal bovine serum (FBS) (Corning, New York, USA), 1 *μ*g/mL insulin, 0.5 mM isobutylmethylxanthine (IBMX), 1 *μ*M dexamethasone, 300 or 500 *μ*M of Tyrosol (TR) (purity ≥ 98%; Cayman Chemicals, Ann Arbor, Michigan, USA), or DMSO (for control cells) (Sigma Aldrich, Saint Louis, Missouri, USA) for 2 days. On day 2, the differentiation medium was replaced with DMEM (4.5 g/L glucose) containing 10% FBS, 1 *μ*g/mL insulin, and 300 or 500 *μ*M of TR or DMSO (for control cells) until day 4. Then, the medium was replaced with DMEM (4.5 g/L glucose) containing 10% FBS and 300 or 500 *μ*M of TR or DMSO (for control cells) changed every 2 days until day 10 [18].

### 2.2. Oil Red O Staining

The Oil Red O staining protocol was modified by Rizzatti et al. [19]. Differentiated cells were washed with phosphate buffer saline (PBS, pH 7.4, Sigma Aldrich) and fixed with 4% formalin (Sigma Aldrich) for 20 minutes (min) at room temperature. Then, cells were washed with double distilled water and then incubated with 60% isopropanol for 5 min at room temperature (Sigma Aldrich). Subsequently, cells were stained with Oil Red O solution (0.5 g/L, Sigma Aldrich) for 30 minutes (min) at room temperature and then washed exhaustively with double distilled water. Pictures were taken using an optical microscope. Moreover, Oil Red O dye retained in the cell was dissolved by pure isopropanol and quantified at 490 nm by using a microplate reader.

### 2.3. Triglyceride Quantification

Triglycerides (TG) were quantified by using the Triglyceride Colorimetric Assay Determination Kit (Cayman Chemicals), according to the manufacturer's protocol. Once TG content was measured, it was then normalized for mg of proteins and reported as mg of TG per mg of cellular protein, according to Roh et al. [20].

### 2.4. Tyrosol Toxicity Assay

Tyrosol toxicity was evaluated during the differentiation protocol at different time points: day 2, day 4, and day 8, by using the (3-(4,5-dimethylthiazol-2-yl)-2,5-diphenyltetrazolium bromide (MTT) assay (Sigma Aldrich) according to the manufacturer's protocol.

### 2.5. Cell Proliferation Assay

Cell proliferation was analyzed by 0.5% Trypan blue staining exclusion (Sigma Aldrich). Viable cells were calculated as the percentage ratio of the number of unstained cells relative to the total cells counted.

### 2.6. Gene Expression Analysis by Real-Time PCR

Gene expression analysis was performed as previously reported [21]. Briefly, total RNA was isolated using the TRIzol reagent (Thermo Scientific). Two and one-half micrograms of total RNA was reverse transcribed into cDNA using a High-Capacity cDNA Archive Kit (Applied Biosystems, Foster City, CA). Inventories under patent primers for leptin, aP2, TNF*α*, IL6, UCP1, and actin were purchased from Applied Biosystems. The relative expression was calculated using the comparative ∆∆CT method, and the values were expressed as 2^-∆∆CT^.

### 2.7. Western Blot Analysis

Western blot analysis was performed as previously described by Pacifici et al. [21]. Briefly, cells were lysed at 4°C in HNTG lysis buffer (1% TritonX-100, 50 mM HEPES, 10% glycerol, 150 mM NaCl, and 1% sodium deoxycholate) supplemented with Phosphatase Inhibitor Cocktail 2 and 3 (Sigma Aldrich) and protease inhibitor cocktail (Sigma Aldrich). Then, proteins were separated on 4-12% SDS polyacrylamide gels (Bio-Rad Laboratories, MI, Italy) and then transferred electrophoretically to nitrocellulose membranes, using the Trans-Blot Turbo System (Bio-Rad Laboratories). The immunoreactive bands were visualized using the Enhanced Chemiluminescence (ECL) kit (GE Healthcare, Little Chalfont, UK) according to the recommendations of the manufacturer. The membrane was exposed to the ChemiDoc System (Bio-Rad Laboratories) and analyzed using Image Lab Software (Bio-Rad Laboratories). Antibodies specific for PPAR*γ*, CEBP*α*, SREBP1c (Novus Biological, Centennial, Colorado, USA), Glut-4, SOD2, AMPK-*α*, phospho-AMPK-*α* (Thr172), HSL, phospho-HSL (Ser565), ATGL (Cell Signaling Technology, Danvers, Massachusetts, USA), Sirt1 and phospho-ATGL (Ser406) (Abcam Cambridge, UK), p21, cyclin D3, and vinculin (Santa Cruz Biotechnology, Santa Cruz, CA, USA) were used.

### 2.8. ROS Evaluation

Reactive Oxygen Species (ROS) levels were measured by using the MitoSOX Red dye (Thermo Fisher Scientific) following the manufacturer's protocol. The quantification of dye fluorescence was assessed by cytofluorimetric analysis.

### 2.9. Cell Cycle Analysis

Cell cycle analysis was performed in fully differentiated 3T3-L1 treated both with TR 300 and 500 *μ*M, modifying the protocol by Drira et al. [16]. Cells were trypsinized, centrifuged, and fixed with ethanol 70% for 30 min. Then, cells were centrifuged, stained with Propidium Iodide solution, and incubated at +4°C for 45 min. Subsequently, cytofluorimetric analysis was performed.

### 2.10. Statistical Analysis

Data were analyzed using Prism 5 (GraphPad, La Jolla, CA) and expressed as the mean ± standard error (SEM). Statistical significance was determined with Student's *t*-test when two experimental groups are present. For more than two groups, statistical evaluation of the data was performed using the ANOVA test, followed by Bonferroni's post hoc test with *p* < 0.05 considered significant.

## 3. Results

### 3.1. TR Inhibits 3T3-L1 Differentiation

**Figure 1:**
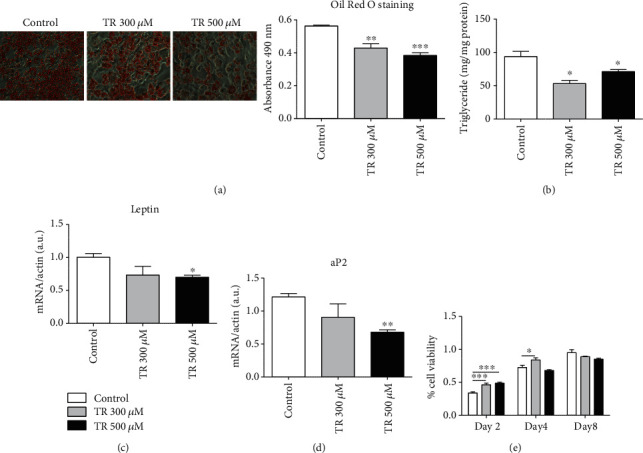
Oil Red O staining and relative triglyceride content in 3T3-L1 adipocytes. Cells were seeded at a density of 1.6 × 10^4^ cells/well in a 24-well plate. Differentiation was induced with or without TR up to day 10. Cells were, then, stained with Oil Red O, and the lipid droplet storage was visualized in optical microscopy and quantified by measuring absorbance (a). Relative triglyceride (TG) content was also evaluated and normalized to mg of protein (b). Leptin (c) and aP2 (d) levels were also evaluated by qRT-PCR. Cell viability was assessed by performing an MTT assay (e). Results are expressed as the mean ± SEM. ^∗^*p* < 0.05, ^∗∗^*p* < 0.01, and ^∗∗∗^*p* < 0.001. Graphs illustrate three different experiments conducted separately.

To investigate whether TR might inhibit adipocyte maturation, differentiation of 3T3-L1 was induced and the intracellular storage of lipids was assessed by performing Oil Red O staining on day 10. Treatment with TR significantly reduced lipid accumulation at both 300 and 500 *μ*M, in a dose-dependent manner, compared with control cells (Figure 1(a)). This result was confirmed by measurement of Oil Red O absorbance at 490 nm. Lipid accumulation in 3T3-L1 was significantly reduced by 20% and 30% following 300 and 500 *μ*M of TR treatment, respectively, compared to the control (*p* < 0.01 and *p* < 0.001, respectively). To further confirm the reduction in intracellular lipid accumulation, and then adipocyte maturation, the cellular content of triglycerides (TG) was measured. A significant decrease in intracellular TG amount was evident at 300 *μ*M than 500 *μ*M of TR (*p* < 0.05), compared to differentiated control cells (Figure 1(b)). Moreover, inhibition in adipocyte differentiation was also validated by measuring leptin and aP2 levels, specific markers of adipose tissue differentiation status and inflammatory state [22, 23]. TR at 500 *μ*M significantly decreased both levels of leptin and aP2, confirming the effect on TR as an antiadipogenic compound (Figures 1(c) and 1(d)). Moreover, we also tested whether TR reduces adipogenesis by blunting cell viability during the differentiation protocol. As showed in Figure 1(e), TR did not reduce cell viability, further confirming its direct effect on adipogenesis. Taken together, all these results highlight the relevant role of TR in reducing adipocyte differentiation that may greatly contribute to a lower adipose cell mass accumulation.

### 3.2. TR Blunts Adipogenesis by Controlling CEBP/*α* and Sirt1/PPAR*γ* Pathways

**Figure 2:**
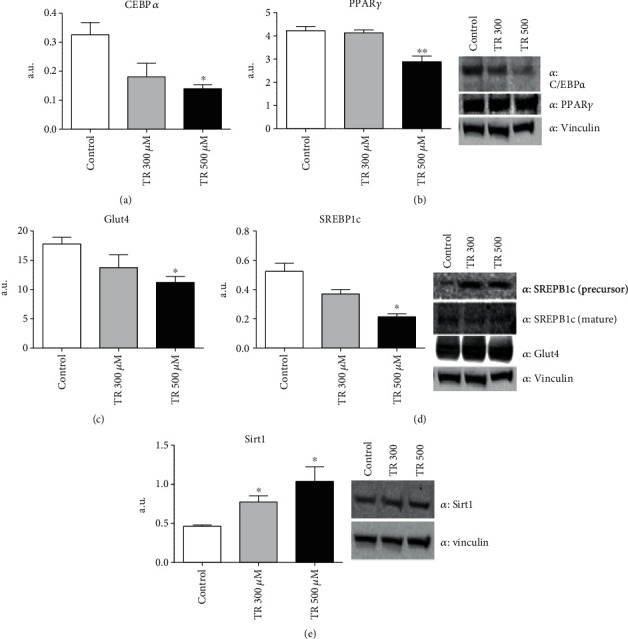
Effect of TR on adipogenic factors. Cells were seeded at a density of 8 × 10^4^ cells/well in a 6-well plate. Differentiation was induced with or without TR up to day 10. Protein steady-state levels of CEBP1/*α* (a), PPAR*γ* (b), Glut4 (c), SREBP1c (d), and Sirt1 (e) were evaluated by Western blot analysis. Results are expressed as the mean ± SEM. ^∗^*p* < 0.05, ^∗∗^*p* < 0.01. Graphs illustrate three different experiments conducted separately.

In order to explore the molecular mechanisms beyond the antiadipogenic effect of TR, we evaluated protein expression levels of CEBP/*α*, PPAR*γ*, Glut4, and SREBP1c, as key regulatory factors of adipogenesis [24–26]. As expected, after 10 days, 500 *μ*M of TR treatment during cellular differentiation significantly reduced expression of both CEBP/*α* (*p* < 0.05) and PPAR*γ* proteins (*p* < 0.01), compared to control cells (Figures 2(a) and 2(b)). Similarly, a decreased expression of Glut4 and SREBP1c was observed in cells treated with 500 *μ*M (*p* < 0.05) of TR at the latest stage of differentiation (Figures 2(c) and 2(d)). These findings suggest that TR blunts adipogenesis by impairing CEBP/*α* and PPAR*γ* pathways.

Sirt1 is a major regulator of lipid metabolism and adipogenesis. In particular, Sirt1 blunts adipogenesis by impairing PPAR*γ* expression [27]. In agreement with these evidences, a significant enhancement in Sirt1 expression was present following TR administration at 300 than 500 *μ*M (*p* < 0.05), compared to untreated cells, suggesting that the antiadipogenic effect of TR may be mediated by the Sirt1/PPAR*γ* pathway.

### 3.3. TR Exerts Both Anti-inflammatory and Antioxidant Effects

**Figure 3:**
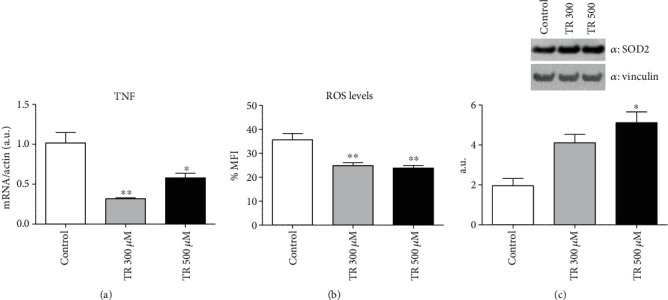
Anti-inflammatory and antioxidant effect of TR. Cells were seeded at a density of 8 × 10^4^ cells/well in a 6-well plate. Differentiation was induced with or without TR up to day 10. mRNA levels of TNF*α* (a) were evaluated by qRT-PCR. (b) ROS levels were assessed by using MitoSOX Red followed by cytofluorimetric analysis; (c) SOD2 levels were evaluated by performing Western blot analysis. Results are expressed as the mean ± SEM. ^∗^*p* < 0.05, ^∗∗^*p* < 0.01, and ^∗∗∗^*p* < 0.001. Graphs illustrate three different experiments conducted separately.

Adipogenesis is characterized by an enhanced proinflammatory state [28]. In agreement with reported previous results on reduced adipogenesis mediated by TR administration, we also evaluated whether TR reduces the proinflammatory state, typical of adipocyte differentiation. In particular, we examined TNF*α* mRNA levels in fully differentiated adipocytes treated with TR. TNF*α* levels were significantly reduced by TR at both 300 and 500 *μ*M (*p* < 0.01 and *p* < 0.05, respectively) compared to control cells (Figure 3(a)).

Oxidative stress and increase in ROS production are another typical feature of adipogenesis and obesity [28]. Based on this evidence, we measured ROS production in mature adipocytes following TR administration. In TR-treated cells, ROS levels were significantly reduced at both 300 and 500 *μ*M (*p* < 0.01), compared to control cells (Figure 3(b)). In association with the decrease in oxidative stress, enhanced levels of the antioxidant enzyme, the Superoxide Dismutase 2 (SOD2), were observed in 500 *μ*M TR-treated cells, suggesting, at least in part, a direct association with the observed blunt in ROS production (*p* < 0.01) (Figure 3(c)). TR may contribute to improving the altered systemic condition caused by obesity through its anti-inflammatory and antioxidant role.

### 3.4. TR Irreversibly Inhibits Adipogenesis by Acting through Proliferation and Differentiation Processes

**Figure 4:**
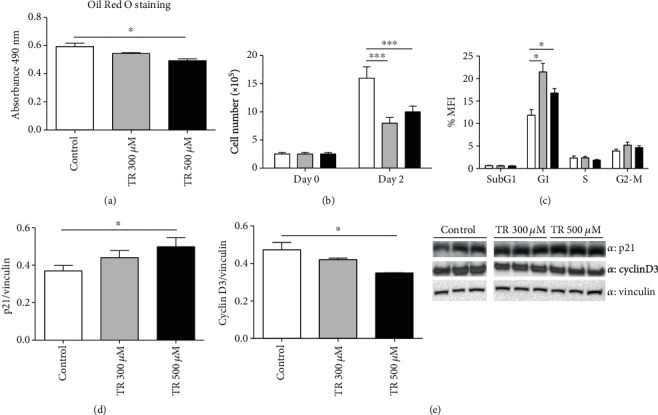
TR effect on MCE and irreversibility of adipogenesis impairment in 3T3-L1. Cells were seeded at a density of 1.6 × 10^4^ cells/well in a 24-well plate. (a) Differentiation was induced at day 0 with or without TR up to day 2, and then, fresh medium without TR was changed up to day 10. Oil Red O staining at day 10 was performed, and lipid content was quantified by measuring absorbance. (b) Cell proliferation was assessed by using the Trypan blue exclusion assay at day 0 and day 2 (representing the MCE). (c) Cell cycle analysis was performed by using Propidium Iodide followed by cytofluorimetric analysis. (d, e) Cell cycle arrest was evaluated by measuring steady-state levels of p21, and cyclin D3 by performing Western blot analysis. Results are expressed as the mean ± SEM. ^∗^*p* < 0.05 and ^∗∗∗^*p* < 0.001. Graphs illustrate three different experiments conducted separately.

We investigated whether the inhibitory effect of TR on adipogenesis might be reversible by treating cells with TR (300 or 500 *μ*M) from day 0 to day 2 (early stage of differentiation). Subsequently, TR was removed, and fresh differentiation medium was administered up to day 10. As shown in Figure 4(a), by Oil Red O staining, a slight but significant inhibition of cellular differentiation was observed after treatment with 500 *μ*M TR (*p* < 0.05), compared to untreated cells, suggesting that TR irreversibly blunts cell adipogenesis by acting at the early stage of differentiation. Since we previously observed a reduction in preadipocyte viability after TR administration, we then evaluated whether TR acted on cell survival and proliferation during the mitotic clonal expansion (MCE). Thus, cell differentiation was induced, and TR was added from day 0 to day 2. The results show that TR at both doses, 300 and/or 500 *μ*M, inhibited MCE, as reported by Trypan blue staining, compared to untreated cells (*p* < 0.001) (Figure 4(b)). Inhibition of cell proliferation was also confirmed by measuring the whole cell cycle analysis. As reported in Figure 4(c), TR administration at both 300 and 500 *μ*M induced cell cycle arrest in G1 phase compared to control cells (*p* < 0.05). To further validate these data, an increase in the steady-state levels of p21 (Figure 4(d)), a well-established inhibitor of cell cycle progression [29], and a reduction in cyclin D3 levels, a promoter of cell cycle progression (Figure 4(e)) [30], were evident in TR-treated cells, compared to control cells (*p* < 0.05). All these findings corroborated the role of TR in reducing adipogenesis by exerting its main action during specific steps of cellular proliferation and differentiation.

### 3.5. TR Treatment Reduces Intracellular Lipid Storage by Triggering Adipose Tissue Browning

**Figure 5:**
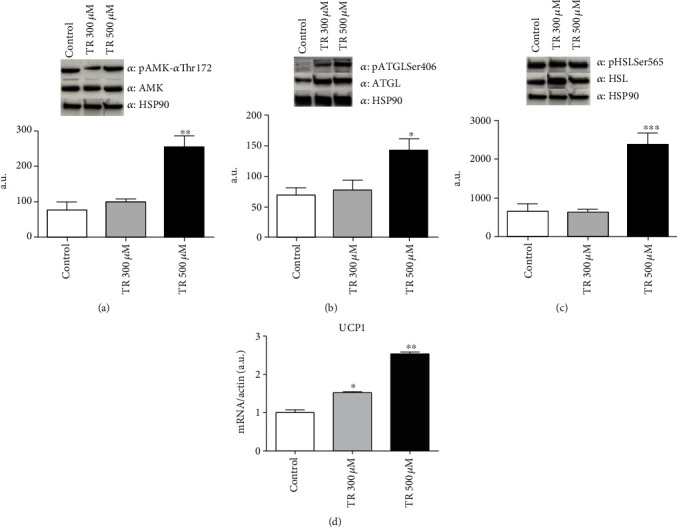
TR induces lipolysis and triggers adipose tissue browning. Cells were seeded at a density of 8 × 10^4^ cells/well in a 6-well plate. Differentiation was induced with or without TR up to day 10. Protein steady-state levels of phospho-AMPK (a), phospho-ATGL Ser406 (b), and phospho-HSL Ser565 (c) were evaluated by Western blot analysis. mRNA levels of UCP1 (d) were evaluated by qRT-PCR. Results are expressed as the mean ± SEM. ^∗^*p* < 0.05, ^∗∗^*p* < 0.01, and ^∗∗∗^*p* < 0.001. Graphs illustrate three different experiments conducted separately.

Given the established role of AMP-Activated Protein Kinase (AMPK) as an adipose tissue sensor of the intracellular energy state, and based on its antiadipogenic role [31, 32], to better understand the mechanistic process of TR-mediated antiadipogenic effect, we investigated the AMPK activation in this process. As reported in Figure 5(a), TR 500 *μ*M determined a significant increase in AMPK-*α* phosphorylation at threonine-172 (Thr172), following 3T3-L1 cell differentiation compared to control cells (*p* < 0.01).

Once activated, AMPK phosphorylates Adipose Triglyceride Lipase (ATGL) at serine-406 (Ser406) leading to an increase of lipolysis in adipose tissue [33, 34]. We examined ATGL activation in 3T3-L1 adipocytes upon treatment with TR, and we observed an enhancement in the phosphorylation of ATGL at Ser406 after differentiation (*p* < 0.05) (Figure 5(b)). We then analyzed the phosphorylation at Ser565 of hormone-sensitive lipase (HSL), another key factor of lipolysis regulated by AMPK [35]. According to AMPK activation, HSL phosphorylation increased following TR 500 *μ*M administration (*p* < 0.001) (Figure 5(c)). Intriguingly, the activatory phosphorylation of AMPK (Thr172) following TR treatment and the subsequent increase in ATGL-mediated lipolysis promote uncoupling protein 1 (UCP1) expression leading to the initial step of adipose tissue browning [36]. Accordingly, both TR 300 and 500 *μ*M enhanced the expression levels of UCP1 in fully differentiated 3T3-L1 (*p* < 0.05 and *p* < 0.001, respectively) (Figure 5(d)), suggesting that TR administration may reduce adipogenesis by promoting adipose tissue browning.

## 4. Discussion

In the present study, we reported the beneficial effect of Tyrosol on reducing adipogenesis and proliferation of murine preadipocyte cell line 3T3-L1. In particular, TR administration significantly reduced adipogenesis by inhibiting the CEBP/*α* and regulating Sirt1/PPAR*γ* pathways. Moreover, treatment with TR at specific doses determined a significant reduction in markers of inflammation and oxidative stress, which are typical processes related to complications of obesity, and may be also helpful during cardiometabolic rehabilitation following cardiometabolic alterations due to obesity. Hence, we reported that TR permanently reduced adipogenesis by blunting cell proliferation and differentiation, which may significantly lead to a decrease in fat mass accumulation and contribute to preventing adipose dysfunction in the context of obesity. Finally, we demonstrated that TR effect on adipogenesis is also directly mediated by activation of AMP kinase, which allows switching adipogenesis into adipose tissue browning, as per the UCP1 levels present after TR treatment.

Several studies reported that bioactive compounds, such as quercetin and epicatechin, by reducing preadipocyte viability (increasing cell death), may contribute to reducing adipose mass [37–39]. The action of these dietary stilbenes and flavonoids in preventing preadipocyte accumulation was more related to their abilities in counteracting the proaggregate function of oxidative stress. In the present study, we demonstrated that TR administration significantly decreases 3T3-L1 viability. Therefore, we may speculate that TR induces direct damage on preadipocytes that may contribute to reducing cell differentiation, adipogenesis, and total fat mass content. This hypothesis is further supported by the effect of *Rhodiola crenulata*, containing TR as a major bioactive phenolic compound, on the inhibition of the activities of proline dehydrogenase (PDH) and glucose-6-phosphate dehydrogenase (G6PDH) [17]. PDH and G6PDH activities are essentials for lipid metabolism related to the cellular structure and survival [40].

In line with our findings, besides inhibition of cell viability, previous studies reported that polyphenols may regulate adipose mass by blunting adipogenesis [10]. Association of resveratrol and quercetin significantly reduced lipid accumulation during 3T3-L1 differentiation [10] through a decrease in CEBP/*α* and PPAR*γ* gene expression [10]. Similar evidence on the decline in CEBP/*α* and PPAR*γ* gene expression was reported in association with impairment in 3T3-L1 differentiation following resveratrol administration [41]. Here, we also observed a reduction in total lipid accumulation on preadipocytes after TR administration, in association with a significant decrease in CEBP/*α* and PPAR*γ* protein expression. PPAR*γ* and CEBP/*α* are the main transcriptional factors for numerous genes during adipocyte differentiation [42]. Among those, Glut4 levels increase during adipogenesis [26]. We demonstrated an increase in Glut4 expression levels during 3T3-L1 differentiation, although a significant reduction of Glut4 levels was observed on day 10 in TR-treated cells. Glut4 is the transporter responsible for glucose uptake by adipocytes; therefore, its activity is pivotal for adipose tissue growth, while lower physiological levels of this transporter may result in prevention of obesity and diabetes [43]. It is worthy to note that downregulation of Glut4-related pathways is linked with increased risk for insulin resistance [44]. However, this association is mainly reported at the skeletal muscle level that accounts for ~80% of glucose uptake after food intake. Our results in adipocytes may not be detrimental since reduction of glucose uptake at adipose tissue levels may be counteracted at the muscle level. SREBP1c represents an important adipogenic regulator factor, directly controlled by CEBP/*α* [45], which increases adipogenesis by inducing PPAR*γ* activation [46]. In our experimental model, SREBP1c levels are significantly decreased in the latest stage of differentiation, after TR administration. Similar findings were reported on preadipocytes following administration of HT and oleuropein, two relevant bioactive compounds present on EVOO [16]. In fact, HT has been indicated among the most powerful antioxidants in the context of metabolic syndrome (body weight/adiposity, dyslipidemia, hypertension, and hyperglycemia/insulin resistance) and associated complications (oxidative stress and inflammation) [47]. However, some differences exist between HT and TR, especially in terms of nutrient concentration, such as in EVOO, where TR is significantly more present [48]. The presence of TR has been also described in red wine and green tea besides EVOO [49]. However, to the best of our knowledge, no data are available so far on the direct role of TR in adipogenesis and related diseases, and the present study is the first in presenting these findings.

Recently, adipogenesis has been demonstrated to be regulated by Sirt1. Sirt1 belongs to the 7 sirtuins, a class III histone deacetylase regulating aging processes and metabolic homeostasis [50]. In particular, Sirt1 deletion or inactivation leads to an increase in adipose tissue and metabolic dysfunction [51]. It has been demonstrated that knockdown for Sirt1 in adipocytes determines an increase in PPAR*γ* levels, which promotes adipogenesis [52], while Sirt1 overexpression in 3T3-L1 blunts adipogenesis by reducing PPAR*γ* expression, and decreases fat accumulation in mature adipocytes [27]. These evidences suggest that the Sirt1 controls adipogenesis in a PPAR*γ*-related way; however, a relevant role of CEBP/*α* in regulating Sirt1 expression during adipogenesis has also been reported yet [53]. Here, we showed that TR administration significantly increased Sirt1 expression, with a subsequent reduction in PPAR*γ*, suggesting that TR antiadipogenic effect may be mediated, at least in part, by the Sirt1 pathway.

Since all the mechanisms previously described, especially Sirt1 and PPAR*γ*-mediated pathways, are strongly associated with inflammation and oxidative stress in metabolic diseases [54], we evaluated markers of these processes in preadipocytes after TR treatment. The reduction of TNF*α*, which is a proinflammatory cytokine [55], is in line with other studies demonstrating the anti-inflammatory role of TR mediated at different levels, including COX-2 expression [56]. Similarly, reduction in ROS production and increase in SOD2 expression, an endogenous antioxidant defense that is significantly decreased in obesity [57], after TR treatment, are in line with the antioxidant activities of the enzymes present in EVOO [56]. These evidences suggest the hypothesis of its possible use as a therapeutic option against obesity, since inflammation and oxidative stress in mature adipocytes represent the main mechanisms leading to major complications, such as diabetes and cardiovascular events [58].

To investigate at which differentiation stage TR exerts its antiadipogenic effect, we tested the association between TR and mitotic clonal expansion (MCE), as a mandatory stage for successful adipogenesis [59]. TR administration during MCE significantly impaired adipogenesis by blunting adipocyte proliferation and by inducing cell cycle arrest, as further confirmed by both the increase in G1 phase and the steady-state level of p21. Based on these results, it is possible to state that TR acts on these cellular processes in a similar manner and magnitude of HT and oleuropein, as previously reported [16]. Other evidences on cocoa polyphenol extract and their role in suppressing adipogenesis during MCE, and in inducing cell cycle arrest during preadipocyte (3T3-L1) differentiation [60], further support the present findings. Treatment with TR significantly reduces cyclin D3 levels in our cellular models. Cyclin D3 promotes adipogenesis through the induction of PPAR*γ* expression [61]. Consistently, it has been shown that cyclin D3 knockdown mice reduced intracellular lipid accumulation in association with decreased levels of PPAR*γ* [61]. Therefore, taken together, our data suggest that TR reduces adipogenesis mainly throughout the inactivation of PPAR*γ*-mediated pathways.

Finally, to go in deep in investigating the mechanistic processes beyond TR effect, we evaluated the modulation of several factors linked to lipolysis. These factors mainly require AMPK for its activation, which ultimately leads to fatty acid release. Furthermore, lipolysis requires also the activation of lipases, such as HSL and ATGL [33]. Upon treatment of adipocyte cultures with TR, we observed that TR induces AMPK activation in association with the increase in ATGL phosphorylation at Ser406, which enhances its hydrolase activity. Moreover, TR also stimulated HSL activation, suggesting that TR specifically affects ATGL function in the adipocyte. The activation of this pathway might explain the reduction in intracellular lipid droplet size, as well as the upregulation of brown adipocyte marker (UCP1) in cell cultures following TR administration, in a dose-dependent manner. In agreement, Ahmadian et al. suggested that ATGL hydrolase activity within adipocytes may lead to UCP1 expression promoting adipose tissue browning [62]. UCP1, in fact, represents the hallmark of brown adipose tissue (BAT) and is a reliable marker of browning of white adipose tissue (WAT), which has been shown to protect from obesity and type 2 diabetes [63]. In accordance with our results, it has been reported that a natural flavonoid protects from altered adipogenesis and obesity by inducing browning of WAT [63]. Thus, our data suggest that the proposed antiadipogenic effect of TR may be mediated by activation of the AMPK-ATGL-UCP1 pathway, leading to browning of WAT. However, further studies are needed to corroborate the present results.

Strengths of this study include the use of two different dosages of TR, tests performed in differentiated and not differentiated cells, and a follow-up time of 10 days that allowed catching differences between cellular groups. The main limitation to acknowledge is related to the impossibility of controlling and measuring all potential molecular pathways activated by TR to control adipogenesis. Moreover, it is important to highlight that this is a study only conducted *in vitro* in a single cellular line with high concentration of TR. Therefore, conclusions should be taken with caution and may not be conclusive since further *in vivo* studies with mice fed a high-fat diet are mandatory to prove the concept.

## 5. Conclusions

**Figure 6:**
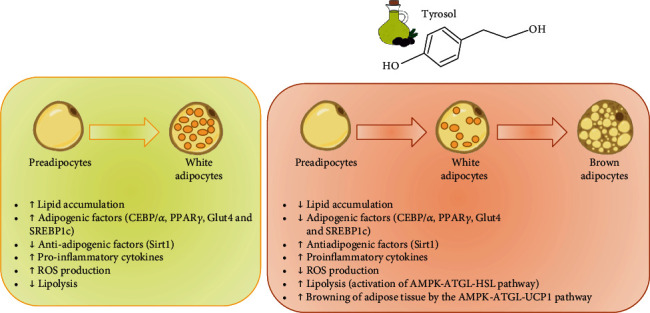
Schematic representation of TR effect on 3T3-L1 preadipocytes.

In the present study, we report a direct antiadipogenic effect of TR as the main EVOO bioactive compound. The TR beneficial role in adipogenesis is exerted by the inhibition of adipocyte differentiation and proliferation mainly through modulation of PPAR*γ*-related pathways and then the inhibition of inflammation and oxidative stress and by switching WAT to BAT (Figure 6). Therefore, a diet with nutrients containing TR, especially after validation of the present data with *in vivo* studies conducted on mice fed a high-fat diet, may be proposed for its potential therapeutic application for the prevention of obesity and to improve quality of life.

## Data Availability

All data are available to the readers by directly contacting the corresponding author in the email reported on the first page.
